# Restoring SMN Expression: An Overview of the Therapeutic Developments for the Treatment of Spinal Muscular Atrophy

**DOI:** 10.3390/cells11030417

**Published:** 2022-01-26

**Authors:** Tejal Aslesh, Toshifumi Yokota

**Affiliations:** 1 Neuroscience and Mental Health Institute, Faculty of Medicine and Dentistry, University of Alberta, 116 St. and 85 Ave., Edmonton, AB T6G 2E1, Canada; aslesh@ualberta.ca; 2 Department of Medical Genetics, Faculty of Medicine and Dentistry, University of Alberta, 116 St. and 85 Ave., Edmonton, AB T6G 2E1, Canada; 3 The Friends of Garret Cumming Research and Muscular Dystrophy Canada HM Toupin Neurological Science Research Chair, 8812 112 St., Edmonton, AB T6G 2H7, Canada

**Keywords:** spinal muscular atrophy (SMA), *survival of motor neuron 1* (*SMN1*), *SMN2*, SMN protein, antisense oligonucleotide (AON), nusinersen, gene therapy, onasemnogene, risdiplam, small molecule

## Abstract

Spinal muscular atrophy (SMA) is an autosomal recessive neurodegenerative disorder and one of the most common genetic causes of infant death. It is characterized by progressive weakness of the muscles, loss of ambulation, and death from respiratory complications. SMA is caused by the homozygous deletion or mutations in the survival of the motor neuron 1 (*SMN1*) gene. Humans, however, have a nearly identical copy of *SMN1* known as the *SMN2* gene. The severity of the disease correlates inversely with the number of *SMN2* copies present. *SMN2* cannot completely compensate for the loss of *SMN1* in SMA patients because it can produce only a fraction of functional SMN protein. SMN protein is ubiquitously expressed in the body and has a variety of roles ranging from assembling the spliceosomal machinery, autophagy, RNA metabolism, signal transduction, cellular homeostasis, DNA repair, and recombination. Motor neurons in the anterior horn of the spinal cord are extremely susceptible to the loss of SMN protein, with the reason still being unclear. Due to the ability of the *SMN2* gene to produce small amounts of functional SMN, two FDA-approved treatment strategies, including an antisense oligonucleotide (AON) nusinersen and small-molecule risdiplam, target *SMN2* to produce more functional SMN. On the other hand, Onasemnogene abeparvovec (brand name Zolgensma) is an FDA-approved adeno-associated vector 9-mediated gene replacement therapy that can deliver a copy of the human *SMN1.* In this review, we summarize the SMA etiology, the role of SMN, and discuss the challenges of the therapies that are approved for SMA treatment.

## 1. Introduction

Proximal spinal muscular atrophy (SMA) is one of the most commonly inherited genetic disorders with a prevalence of 1/11,000 births and a carrier frequency of 1/40 to 1/60 [1]. SMA is mainly characterized by the degeneration of alpha motor neurons located in the anterior horn of the spinal cord. In its most severe form, the disease onset occurs before 6 months of age in infants and leads to weakening of the muscles, loss of bulbar function, compromised breathing, and an inability to sit independently. The proximal muscles of the body are predominantly affected in early onset SMA, leading to a very limited range of mobility [2,3]. Infants also suffer from respiratory distress and ultimately die within 2 years of age [4]. SMA is caused by mutations in the survival of motor neuron 1 gene (*SMN1*), which is majorly involved in the proper functioning of the motor neurons by assisting the assembly of small nuclear ribonucleoprotein complexes (snRNPs) [5]. The translated product SMN is a 30 kDa essential protein that is expressed in almost every cell: in both the nucleus and the cytoplasm. SMN loss from the cellular machinery results in reduced SMN protein expression, leading to a degeneration of motor neurons and progressive muscle weakness and atrophy [6,7,8]. Humans possess a paralog of *SMN1* called *SMN2* caused by the intrachromosomal duplication of 5q13. The centromeric *SMN2* critically differs from the telomeric *SMN1* at the base pair position 840, a C-to-T substitution that ultimately excludes exon 7 from around 90% of the *SMN2* mRNA transcripts [9]. These transcripts lacking exon 7 produce very low levels of SMN protein because they are unstable and therefore rapidly degrade. The severity of the disease correlates inversely with the SMN protein levels and the *SMN2* copy number [10].

It is fortunate that we now have a few approaches that have resulted in FDA approval for the treatment of SMA. Here, we will focus on several approaches to restore SMN, including the use of synthetic nucleic acids called antisense oligonucleotides (AONs) for splice modulation, small molecules, and adeno-associated, vector-mediated gene replacement. At present, there are three approved treatments by the Food and Drug Administration (FDA) that are directed towards increasing the production of SMN protein. The first approved treatment is an AON drug known as nusinersen (brand name Spinraza) that targets a silencer region in the intronic region of the *SMN2* gene, thereby providing stability to the transcripts and preventing their degradation [11]. Nusinersen is approved for all SMA types and is intrathecally administered via lumbar punctures into the cerebrospinal fluid (CSF). Patients receive up to seven injections in the first year of treatment, with subsequent maintenance doses every 4 months. Albeit the first successful breakthrough for SMA treatment has been achieved, the exorbitant treatment costs and adverse side effects still pose significant challenges. Viral-vector-based gene therapy (Zolgensma, onasemnogene abeparvovec-xioi) for SMA was approved by the FDA in 2019 to deliver a functional copy of the SMN cDNA as a one-time intravenous administration to patients below the age of 2 years [12]. The long-term effects of SMN overexpression and immune response to the viral vector have led to questions about the safety of gene therapy for SMA. Besides, patients with late-onset SMA (>2 years) are not eligible for this treatment. Results from clinical trials addressing these concerns are currently awaited. The third approved drug is known as risdiplam (brand name Evrysdi) developed by Roche, PTC Therapeutics Inc. and the SMA Foundation [13]. It is an orally available *SMN2*-directed RNA splicing modifier that received its approval in August 2020 for patients 2 months and older. It has an advantage over nusinersen in terms of avoiding invasive injections all together with no apparent toxicity. The long-term efficacy of risdiplam is yet to be investigated. Table 1 summarizes the current approved treatments for SMA. At present, researchers are also evaluating the possibility of combination therapies to promote improved efficacy and better protection from the debilitating effects of the disease.

In this review, we will discuss in brief the background of SMA, its molecular characteristics, and currently approved treatments to restore SMN expression. We shall also provide insights into alternative treatment strategies that are currently being studied to ameliorate the disease phenotype.

## 2. SMA: Background

SMA was first described by Guido Werdnig in 1891 when he presented a case of muscle weakness in two infant brothers (10 months), followed by seven additional cases that were reported by Johan Hoffmann from 1893 to 1900 [14,15]. The most severe form of SMA also became known as ‘Werdnig-Hoffmann syndrome’. A milder form of SMA (Kugelberg–Welandar disease) was briefly described in the 1950s by Wohlfart, Fez, and Eliasson, and in depth by Kugelberg and Welandar [15]. The patients were ambulatory and had prolonged survival. All the cases presented had reported signs of degeneration of the anterior horn cells of the spinal cord, coupled with proximal muscle weakness that affects the axial, intercostal, and bulbar musculature [14,15]. The next half of the 20th century characterized the variability of the severity of SMA seen in patients. A controversy arose regarding the characterization of SMA as infantile, juvenile, and adult forms because they represented both single and multiple diseases [14]. This eventually led to the formation of a classification scheme at the International Consortium on Spinal Muscular Atrophy (sponsored by the Muscular Dystrophy Association) in 1991 [16]. This scheme presented three types of SMA based on the highest level of motor function (sitting or standing), along with the age of onset of the disease. Subsequently, a type 4 SMA was added to include adult-onset cases, and a type 0 for patients with prenatal onset of SMA, and death within a few weeks [14]. This SMA classification scheme provided a useful prognosis of the disease and is still relevant today for treatment. Table 2 summarizes the classification of SMA based on the number of copies of *SMN2*.

## 3. SMA: Molecular Characteristics

**Figure 1:**
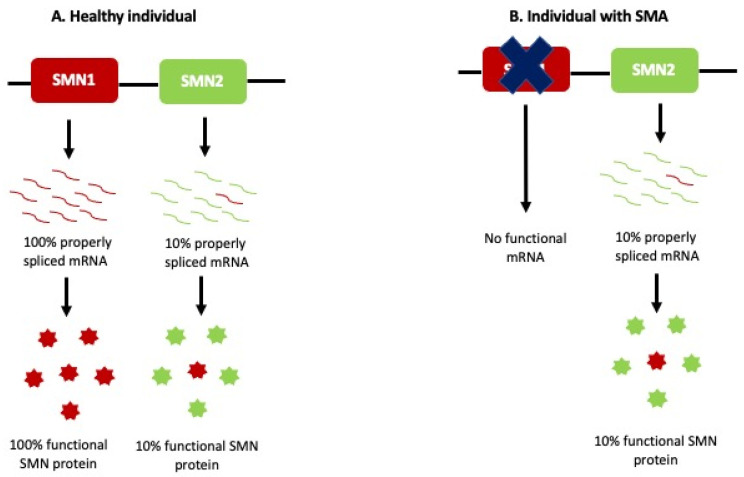
Etiology of SMA. (**A**) *SMN1* can produce 100% properly spliced mRNA, which is translated to functional SMN protein in healthy individuals. *SMN2* can produce only 10% functional mRNA transcripts, while the remaining 90% *SMN2* transcripts lack exon 7 and are rapidly degraded. (**B**) Patients with SMA do not have *SMN1* and rely on the 10% SMN protein produced by *SMN2*. This cannot compensate for the loss of *SMN1*.

Despite the varying phenotypes and severity seen in SMA patients, the causal genetic factor has been localized to the same locus chromosome 5q11.2–13.3, which led to the identification of the *SMN1* gene as the SMA-causing gene in 1994 by Melki and colleagues [17,18]. *SMN1* occupies a 500 kb duplication region that arose from a human-chimpanzee primate ancestor [19]. Due to a high number of repetitions, this locus is unstable and is deleted in the majority of SMA patients [20]. A divergence event led to the formation of the human-specific *SMN2* gene, a paralog of the *SMN1* gene. The major difference between these paralogs is a C-to-T transition in exon 7 of the *SMN2* gene, resulting in unstable mRNA transcripts that are rapidly degraded. The SMN protein translated from such *SMN2* transcripts cannot fully compensate for the loss of *SMN1* in SMA patients as seen in Figure 1.

SMN protein, encoded by the *SMN1* and *SMN2* genes, is ubiquitously present in every cell of the body with various identified functions, including transcriptional regulation, telomerase regeneration, and cellular trafficking. SMN protein (38 kDa; 294 amino acids long) is evolutionarily conserved in humans. SMN protein forms a complex with Gemins2–8 in the cytoplasm and in discrete nuclear bodies called gems [21,22]. The complex is responsible for the biogenesis of spliceosomal snRNPs and pre-mRNA splicing [23]. The SMN complex is disrupted in the cells and tissues of SMA patients, which in turn leads to widespread splicing anomalies [24]. Additionally, recent studies have also illustrated the role of SMN in diverse functions such as autophagy, cellular homeostasis, signal transduction, DNA repair, and recombination [25]. The ubiquitous SMN protein maintains higher levels of expression in the gestational and neonatal stages of development beyond the neuromuscular system, followed by a decline with age [20]. However, the motor neurons of the spinal cord continue to express high SMN levels throughout their lifespan and are hence the most susceptible to SMN loss in the disease phenotype [26]. The exact cause of motor neuron death and its link to SMN loss remains unclear and unanswered.

SMN protein also plays an important role at the neuromuscular junctions (NMJs). NMJs serve as points of contact or synapses between motor neuron nerves and muscle fibers. Studies have shown SMN to be localized in the pre-synaptic terminals at the NMJ, and it has a vital role in recruiting and transporting RNA transcripts along axons during the process of axonogenesis [27]. Reduced levels of SMN impair the normal maturation process of NMJs and also lead to neurotransmission defects in mice. Cellular defects of the NMJs precede symptoms in SMA [28]. SMN protein plays a role in the assembly of U7 snRNPs, which in turn facilitate the 3′-end processing of replication-dependent histone mRNAs that provide integrity to NMJs [29]. These defects may be linked to muscle weakness and motor neuron death, although further evidence is required. 

## 4. Therapeutic Targets

### 4.1. SMN-Dependent Therapies

SMA phenotype arises from a drastic reduction in SMN levels in the cells in both the central and the peripheral nervous systems (CNS, PNS). Since SMN levels decline with age in most species, the time of intervention plays a crucial role in determining the success of treatment strategies. The *SMN2* gene, which is unique to humans, provides a promising opportunity to increase SMN levels by modifying the splicing of exon 7 or increasing the transcription of *SMN2* [20].

#### 4.1.1. Antisense Oligonucleotide (AON)-Based Treatment for SMA

An element located downstream of the 5′ splice site (ss) of exon 7 of *SMN2* called intronic splicing silencer N1 (ISS-N1) was discovered by Singh et al. [30]. This 15-nucleotide (nt) element showed inhibitory effects on the inclusion of exon 7 during splicing. Deleting or masking the ISS-N1 region by the use of AONs promoted the inclusion of exon 7 in the majority of the *SMN2* transcripts [31]. AONs are single-stranded, DNA-like molecules that are extensively studied to manipulate gene expression by hybridizing to mRNA-splicing factors [32]. AONs are designed complementary to intronic or exonic sequences and can either disrupt or enhance the splicing of the target. In the context of SMA, these AONs targeting ISS-N1 prevent the recruitment of heterogeneous nuclear RNP A1 (hnRNP A1) protein to ISS-N1, thereby enhancing exon 7 inclusion and ultimately incrementing SMN levels [31]. The discovery of ISS-N1 proved to be a milestone in the search for a cure for SMA because this concept applies to most SMA patients. This eliminates the need for personalized treatments for the patients [3]. 

To date, many studies have demonstrated that AONs can promote exon 7 inclusion in *SMN2* transcripts and subsequently increase SMN levels in SMA fibroblasts and several mouse models. In the initial set of studies, AONs with a 2′-O-2-methoxyethyl (MOE) chemistry and a phosphorothioate backbone targeting ISS-N1 enhanced exon 7 inclusion, thereby increasing the SMN levels in cultured fibroblasts as well as in vivo in mice that were intravenously injected with the AON [33]. The main obstacle that hindered the success of AON delivery in vivo in preclinical studies was the failure to penetrate the blood–brain barrier (BBB). This was first overcome by a study led by Williams J et al., who demonstrated that the repeated intracerebroventricular (ICV) administration of another AON chemistry 2′-O-methyl (2′OMe) increased SMN levels in the central nervous system (CNS) tissues in a mouse model SMNΔ7 SMA mice (SMNΔ7^+/+^; SMN2 ^+/+^; Smn^−/−^) [34]. Studies led by Hua et al. and Passini M et al. demonstrated the efficacious delivery of MOE AONs targeting the same ISS-N1 sequence to the brain and the spinal cord using ICV injections [35,36]. However, subsequent studies also highlighted the importance of peripheral SMN restoration using MOE, which contributes to an extended survival [37]. 

With the field of AONs rapidly evolving, a new chemistry with a morpholino ring was conceived by James E. Summerton (Gene Tools) [38]. These AONs, also known as PMO (phosphorodiamidate morpholino oligomer), became widely studied for neuromuscular disorders. PMOs are neutrally charged and have enhanced stability with minimal toxicity. Due to a phosphorodiamidate linkage, these AONs are neutrally charged [39,40]. This provides them with an opportunity to be conjugated with peptides for enhanced delivery and tissue uptake [39,40]. With respect to SMA, Porensky et al. compared the efficacy of morpholino PMO HSMN2Ex7D (−10, −29) using ICV, intravenous (facial vein), or combined (facial vein, ICV) delivery routes at different doses (high—81 μg/g, medium—54 μg/g, low—27 μg/g) and demonstrated a dose-dependent survival that corresponded with increased *SMN2* levels in the *SMN*Δ*7* mouse model [41]. They observed striking results with single ICV administration of the PMO, but the results were similar in the intravenous and combined routes. With the flourishing data presented by multiple groups from 2009 to 2012, another group presented a study where they compared three PMOs with varying lengths (18 mer, 20 mer, and 25 mer sequences) at two doses—20 μg/g and 40 μg/g—using ICV, intravenous (IV), or two repeated systemic injections (intravenous with subcutaneous/intraperitoneal) [42]. This group resorted to using the severe Taiwanese mouse model (FVB.Cg-Tg(SMN2)2Hung Smn1tm1Hung/J) created by Hsieh-Li and colleagues [43]. What was interesting to note was that a single intravenous dose of 40 μg/g of the longer PMO (PMO25) extended survival better than both the single ICV and the repeated systemic injections at the same dose. Both groups (Porensky et al. and Zhou et al.) emphasized the superiority of the morpholino AONs because of lower toxicity, increased SMN levels, and prolonged survival [41,42]. The leaky nature of the BBB was speculated to facilitate the restoration of SMN in the CNS tissues. Though the superiority of the delivery routes remained controversial, Nizzardo et al. investigated different doses and routes of the same 25 mer PMO studied by Zhou et al. in targeting ISS-N1 [44]. Contrary to previous results, they emphasized the need for a combined treatment of ICV and peripheral administration of the morpholinos in the *SMN*Δ*7* mouse model, albeit the survival was similar to previous studies with a single ICV dose [44]. Nevertheless, all the studies highlighted the benefits of PMOs for treatment. The superiority of PMOs is further bolstered by the approval of several PMO-based AONs for the treatment of Duchenne muscular dystrophy (DMD) such as eteplirsen, golodirsen, viltolarsen, and casamirsen [45,46].

As mentioned above, neutrally charged PMOs provide them with an opportunity to be conjugated with cell-penetrating peptides (CPPs) for enhanced delivery [39,40]. CPPs are not something recently discovered; they were identified in the late 1980s [38]. CPPs are less than 30 amino acids long and can be positive, negative, or neutral in charge, which facilitates their ability to enhance the uptake/internalization of the cargo in target cells [47]. The first CPP was a 9-amino-acid sequence expressed in the trans-activator protein (TAT) [48,49]. CPPs conjugated to AONs then became widely studied in the context of neuromuscular disorders to efficiently deliver the AON to the CNS tissues [50,51]. The majority of the CPPs are cationic, making them practically impossible to conjugate to negatively charged AONs [47]. Here is where neutrally charged AONs such as PMOs or peptide nucleic acids (PNAs) come to the rescue. In the context of DMD, arginine-rich peptides conjugated to exon-skipping PMOs significantly increased the dystrophin levels in skeletal tissues in DMD mouse models [52,53,54,55,56]. A novel PMO-internalizing peptide (Pip) delivery technology showed promising uptake in the vital and difficult-to-penetrate cardiac muscles [50,57]. One of the Pip family peptides, called Pip6a, when directly conjugated to a morpholino using systemic delivery, ensured body-wide restoration of SMN levels, prolonged survival, and rescued the phenotype [58]. By making use of advantages of CPPs conjugated to AONs, including those of improved cellular uptake, BBB penetration could be extended to several other neurodegenerative disorders such as Huntington’s disease, amyotrophic lateral sclerosis (ALS), or Parkinson’s disease that need antisense therapy targeting different regions of the brain and the spinal cord [59,60,61,62,63]. 

#### 4.1.2. Nusinersen—First Approved AON for SMA

Seminal studies carried out by Passini et al. in SMA mice demonstrated a successful increase in SMN levels that could further overcome the genetic defects seen in SMA [36]. This alongside several other preclinical studies paved the way for phase 1 clinical trials of nusinersen that were sponsored by Biogen and Ionis Pharmaceuticals [36,64]. To hybridize it to ISS-N1, nusinersen is synthesized as an 18-nucleotide sequence TCACTTTCATAATGCTGG, which disrupts the hnRNP and promotes the inclusion of *SMN2* exon 7 [35,65,66].

Phase I of the clinical trials involved 28 SMA patients between 2 and 14 years of age that received single intrathecal administration via a lumbar puncture into the CSF [66]. Data from phase I indicate that the drug could be administered without serious adverse effects, prompting the drug to move to phase II, which was an open-label, dose-escalation study. A total of 20 infants aged between 3 weeks and 7 months were enrolled in this study. They received either 6 mg or 12 mg of the drug injected intrathecally on days 1, 15, 85, and 253, with follow-up doses every 4 months [11,64,67]. Modest improvements in motor milestones were observed in 16 infants, which further encouraged a phase III. The motor outcomes were measured using the Hammersmith Infant Neurological Exam-Part 2 (HINE-2) and the Children’s Hospital of Philadelphia Test of Neuromuscular Disorders (CHOP INTEND). 

A randomized, double-blinded, phase III study (ENDEAR, NCT02193074) had 121 patients enrolled that received 12 mg of nusinersen administered on days 7, 15, 29, 64, 183, and 302 [11]. The primary endpoints included achieving motor milestones and event-free survival. The interim results analysis coupled with data from other trials led to the approval of nusinersen (brand name Spinraza) as the first drug for all SMA types by the FDA on 23 December 2016. Another phase III, double-blinded, placebo-sham-controlled study (CHERISH, NCT02292537) had 126 infants with SMA type 2 administered with 12 mg of nusinersen. They observed a significant increase in the motor milestone scores (Hammersmith Functional Motor Scale-Expanded, HFMSE methodology), which were evaluated for 15 months [11].

A phase II study enrolled 17 pre-symptomatic infants (NURTURE, NCT02386553) who were identified from the pilot newborn screening program or diagnosed because of siblings with SMA. Interim results from this study revealed that nusinersen treatment is most efficacious when administered before the disease onset [68]. A safety evaluation of 4 years of the NURTURE study and an open-label, phase III extension study (SHINE, NCT02594124) revealed no major safety concerns pertaining to liver toxicity, and the common side effects seen were associated with upper respiratory tract infections and pyrexia [69]. Another study revealed that intrathecal administration of nusinersen to SMA type 2 and 3 patients was well-tolerated and safe despite obstacles such as scoliosis that make the administration challenging [70,71]. 

Nusinersen is a successful treatment story because it caters to all SMA types and ameliorates muscle weakness, revives the motor neuron connection with the muscle, and addresses respiratory impairment to some extent. However, SMA type 1 and 2 patients still rely on assisted respiration from time to time (non-invasive ventilation, NIV) as they cannot respire independently [72]. Nusinersen is currently administered via repeated intrathecal injections at a dose of 12 mg. Intrathecal injections are indeed cumbersome, and it would be advantageous to reduce the frequency of injections by increasing the dose. A randomized, double-blind, interventional phase II-III clinical study called DEVOTE (NCT04089566) is testing higher nusinersen doses in this regard to reduce the injection frequency in the future [73]. The results of this study are not yet out, as it is still ongoing. The success of nusinersen is indeed hindered by the poor penetration of the BBB because of its chemistry, which leads to alternatives such as CPP-conjugated morpholinos, which are now being evaluated to target both systemic and CNS tissues [58]. 

#### 4.1.3. Gene Therapy for SMA

Gene replacement therapy is a direct option for increasing *SMN1* gene expression by delivering the *SMN1* cDNA using a viral vector. The ability of an adeno-associated virus (AAV) to transduce motor neurons, deliver SMA therapeutics, and alleviate symptoms has been demonstrated in pre-clinical and clinical studies for SMA treatment. While Foust et al. intravenously administered a scAAV9 (self-complementary AAV9) vector to deliver SMN, Dominguez et al. used the same scAAV9 to ferry a codon-optimized *SMN1* gene to the same *SMN*Δ*7* mouse model [74,75]. Both groups observed striking improvement in survival, motor activity rescue to almost normal levels, and reduction in motor neuron death [74,75]. Since both groups focused on systemic studies, a couple of studies then resorted to ICV-mediated gene therapy. One of the groups tested the AAV9 vector controlled by the expression of the ubiquitous chicken-β-actin (CBA) promoter and found a dose-dependent increase in the survival in the same mouse model when injected on postnatal day 1 with a median survival of 282 days for a dose of 3.3 × 10^13^ vg/kg [76]. Based on the extensive dosing studies in mice and non-human primates, they demonstrated that a lower dose of 1 × 10^13^ vg/kg administered intrathecally in cynomolgus monkeys increased motor neuron transduction, but this was reduced in peripheral tissues [76]. Another group showed that a combined administration (ICV+IV) of scAAV9 carrying a codon-optimized *SMN1* sequence had no effect on the survival of the SMNΔ7 mice [77].

#### 4.1.4. Onasemnogene Abeparvovec: Approval

Pre-clinical work on SMN gene therapy (AVXS-101, Zolgensma, onasemnogene abeparvovec-xioi) laid the foundation for clinical research in SMA patients using the scAAV9 delivery approach. With the idea of a body-wide treatment approach, a phase I, non-randomized study had 15 patients enrolled who intravenously received either a low dose of 6.7 × 10^13^ vg/kg (*n* = 3) or a higher dose of 2 × 10^14^ vg/kg (*n* = 12) of the AAV9-SMN gene under the control of a hybrid CMV enhancer/CBA promoter (NCT02122952) [78]. The primary outcome was to assess treatment-related toxicity, while the secondary outcome was to determine if treated patients needed respiratory support for ≥16 h per day continuously for ≥2 weeks in the absence of an acute reversible illness, excluding perioperative ventilation or death. The cohort receiving the higher dose also had a rapid increase in the motor milestone score (CHOP-INTEND scale), where most of the patients sat unassisted, rolled over and fed orally, and two of them could even walk independently [78,79,80]. Elevated liver enzymes were attenuated by prednisolone treatment. An interventional phase III, open-label trial (STRIVE, NCT03306277) with 22 SMA type 1 patients (age < 6 months) received an intravenous dose of onasemnogene [12,81]. Out of the 22 patients, 19 completed the study. The primary endpoints were sitting unassisted for 30 s at 18 months of age, and if this was statistically significant, the co-primary endpoint was an event-free survival determined by permanent ventilation (tracheostomy) or ≥16 h per day continuously for ≥2 weeks in the absence of an acute reversible illness. The secondary outcome was the ability to thrive—receiving food orally without mechanical support, the ability to swallow thin liquids, and maintaining weight. Ten of the twenty-two participants developed severe adverse events, while all the 22 participants developed non-serious adverse effects. Fifty percent of the 22 patients were sitting independently, 76% of 21 patients achieved head control, and around 41% of the 22 patients could roll over [81]. 

In addition to intravenous administration of gene therapy, another clinical trial aimed to study the intrathecal administration of onasemnogene as a therapeutic candidate to treat patients with milder SMA (three copies of *SMN2*). This STRONG trial (NCT03381729) was a phase I, open-label study that compared different doses of the gene therapy (6.7 × 10^13^ or 1.2 × 10^14^ or 2.4 × 10^14^ vg/kg) in 51 patients. However, this study was suspended because several safety concerns were independently raised in studies pertaining to toxicity in non-human primates [82]. Results from two separate phase III trials that have enrolled SMA type 1 patients with one or two copies of *SMN2* (NCT03837184) and that have pre-symptomatic patients with two or three copies of *SMN2* (NCT03505099) are currently awaited. These clinical trials will provide us with a better perspective of the efficacy of gene therapy and the appropriate time for an intervention. To date, onasemnogene is approved as a one-time intravenous drug (1.1 × 10^14^ vg/kg) for all SMA types below the age of 2 years [82]. Though this therapy is no less than a miracle for the SMA community, especially type 1 SMA infants, new studies are stirring up the fact that overexpression of SMN, especially in the sensorimotor circuit, can lead to a toxic gain of function [83]. A study showed that systemic administration of an AAV9 variant carrying SMN induced severe toxicity, ataxia, and proprioceptive defects in piglets but no overt motor deficits in non-human primates [84]. Neuronal damage including transcriptomic alterations in the dorsal root ganglion (DRGs), cytoplasmic aggregation of SMN, reduction in the number of motor neurons, and late-onset neurodegeneration were observed following the intrathecal administration of AAV9-SMN [84]. These studies serve as a conceptual framework for the existing treatment to explain unforeseeable adverse effects in the future that may not be necessarily associated with AAV9 [83,84]. Late-onset neurodegeneration may follow the early clinical benefits in SMA patients treated with scAAV9-SMN. Follow-up studies may underscore the addition of ‘turn-on/off’ switches to combat unprecedented circumstances of neuronal toxicity [83]. A long-term follow-up study (NCT03421977) of the completed phase 1 study (NCT02122952) is currently active that will ensure safety monitoring of the patients for up to 15 years. The estimated completion of this study is December 2033. 

#### 4.1.5. Small-Molecule Drugs

To determine whether pharmacologically increasing SMN levels could ameliorate the SMA phenotype, a library of molecules was screened in vitro using an HEK293H (human embryonic kidney) cell line that harbored an *SMN2* minigene [85]. Out of these, three classes of compounds were orally available (SMN-C1, SMN-C2, SMN-C3) that increased the full-length *SMN2* levels, and simultaneously decreased the Δ7 *SMN2* levels in type 1 SMA patients’ fibroblasts [85]. These molecules could also penetrate most of the body tissues and rectify *SMN2* splicing in all the cells of the SMNΔ7 mouse model [85]. Other groups reported additional highly specific orally available molecules coumarin 1, isocoumarin 2, and pyridopyrimidinone derivates that specifically modify *SMN2* splicing both in vitro (patient cells) and in vivo (mouse models) [86,87]. However, both coumarin 1 and isocoumarin clinical developments were halted because of toxicity. One of the compounds from the pyridopyrimidinone series became the first orally active small-molecule *SMN2*-splicing modulator (RG7800, RO6885247)), to enter human clinical trials for SMA [88].

Although not exclusive to SMA, histone deacetylase (HDAC) inhibitors have been shown to activate *SMN2* transcription [89]. Acetylation and deacetylation are important processes in the epigenetic regulation of genes [20]. HDACs deacetylate the chromatin histones, creating a transcriptionally repressed region [20]. Inhibiting this can activate gene expression. Several of these molecules including sodium butyrate, valproic acid, trichostatin A, and sodium phenylbutyrate have shown promising results by increasing SMN levels in vitro and in vivo. However, positive clinical outcomes in SMA patients have not yet been exhibited [90]. To further evaluate the potential of HDAC inhibitors, there is a need to develop molecules that can penetrate the BBB to treat SMA [89]. 

Celecoxib, a selective inhibitor of cyclooxygenase 2 that crosses the BBB, increased SMN protein levels by activation of the p38 pathway in rodents [20]. However, the clinical trials were prematurely halted (NCT02876094). Salbutamol/Ventolin, a beta2-adrenoceptor agonist, is another well-tolerated orally administered molecule that increased full-length *SMN2* expression and incremented SMN protein levels in vitro and in vivo [91]. A phase IIb trial with type 3 SMA patients revealed increased SMN protein levels in patient blood samples along with an improvement in motor function [91]. Further research is required to determine how beta2-adrenergic agonists boost SMN production and improve motor performance. 

#### 4.1.6. Risdiplam (Evrysdi): Small-Molecule Compound for SMA

The discovery of ‘compound **2**’ from the pyridopyrimidinone series by Ratni et al. (RG7800, RO6885247) made its way to clinical trials as the first orally active drug. Single oral doses were administered to healthy males in a single-ascending-dose, double-blinded fashion [92]. Compound **2** was safe and well-tolerated at all doses, with the highest dose being selected as the most efficacious one. A 2-fold increase in SMN protein levels relative to the baseline levels was observed 12 weeks post-administration [93]. However, this trial had to be paused as a precautionary measure because findings from a study in cynomolgus monkeys that was run parallel to this human study revealed preclinical chronic retinal toxicity [92]. The same group characterized and optimized additional molecules in the pyridopyrimidinone series, which gave them a successful compound **1** (risdiplam, RG7916) that led to promising results in vitro in SMA type 1 fibroblasts. The optimization and detailed toxicity assays are entailed in [92]. Additionally, adult C/C allele mice were treated once daily for 10 days at different doses (1, 3 or 10 mg/kg), while SMNΔ7 mice were injected intraperitoneally with risdiplam once daily from postnatal day 3 to 9. Both mouse models demonstrated an increase in SMN levels, an increase in motor neuron number, and increased innervation patterns in the neuromuscular junctions (NMJs). Like compound **2**, risdiplam also exhibited retinal toxicity in monkeys, which implicated a class effect of this series [92]. However, this was followed up by a study of both compounds in pigmented vs. albino rats. They found no evidence of retinal changes in the rats [92]. Risdiplam continued to be studied in clinical trials as it was more efficacious than compound **2**. 

Part 1 of a phase II-III clinical trial FIRE-FISH (NCT02913482) had enrolled 21 type 1 SMA infants between the ages of 1 and 7 months [94]. Part 1 focused on the safety, pharmacokinetics (PK), pharmacodynamics (PD), and the blood SMN protein concentrations, while part 2 focused on the risdiplam dose. The dosing strategy (daily oral administration) followed an escalation pattern starting at either 0.04 mg/kg, 0/08 mg/kg, or 0.2 mg/kg depending on the participant’s age. The dose was adjusted to 0.2 mg/kg within a few months of starting the treatment. This was ultimately adjusted to 0.25. mg/kg when the participant reached 3 years of age. The infants were assessed for the motor milestones based on the CHOP-INTEND and the HINE scale at baseline [93]. The most common serious adverse events included respiratory tract infections, and four infants died from respiratory complications. No retinal toxic effects as seen in cynomolgus monkeys were observed in this human study. Additionally, the infants that survived did not require permanent ventilation at 12 months of age, and 7 of the 21 infants could also sit independently [93]. Part 2 of this study bolstered the clinical benefit of risdiplam for type 1 SMA patients with significant improvements in motor functions beyond 12 months. The primary endpoint was met with 29% of patients sitting independently for more than 5 s [94]. On 7 August 2020, the FDA granted approval to risdiplam, an RNA *SMN2* splicing modifier for the treatment of SMA in ages 2 months and up [13]. 

Another study, SUNFISH (NCT02908685), a phase II-III, randomized, double-blind, placebo-controlled study, enrolled SMA type 2 and 3 patients to test the safety, tolerability, PK, PD, and the efficacy of risdiplam. Similar to the FIREFISH study, this study also comprised two parts—part 1 focusing on exploratory dose-finding for 12 weeks, and a confirmatory part 2 for 24 months. Adolescents and adults in the risdiplam cohort between the ages of 12 and 25 years received either 3 mg or 5 mg for at least 12 weeks, and then once the part 2 dose was selected, the participants were switched to the part 2 open-label study [13]. Participants in the control cohort for this group received a matched placebo for 12 weeks, followed by the dose of risdiplam (3 or 5 mg), and ultimately were moved to the part 2 open-label study. Children aged 2–11 years escalated from either a dose of 0.02 mg/kg to 0.05 mg/kg to 0.15 mg/kg or directly from 0.05 mg/kg to 0.15 mg/kg. Another cohort of this children study received 0.25 mg/kg in part 1 of the study. A total of 2 out of 10 adults that received 5 mg/kg dosing reported adverse effects such as nausea, pneumonia, and vomiting. A total of 1 out of 21 children (2–12 years) that received the 0.15 mg/kg dose exhibited an upper respiratory tract infection. Four out of seven children in the 0.25 mg/kg cohort reported adverse events such as gastroenteritis, dehydration, loss of appetite, upper respiratory tract infection, and chronic respiratory failure. Overall, the results were promising with no major side effects or toxicity presented. Results from another clinical trial, Rainbowfish (NCT03779334), which is focused on pre-symptomatic infants 0–6 weeks of age who were genetically diagnosed with SMA, are currently awaited.

Currently, the approved dose for risdiplam is dependent on body weight and age—0.2 mg/kg/day for patients 2 months–2 years, 0.25 mg/kg/day for patients 2 years and up and less than 20 kg in weight, and 0.5 mg/kg/day for those (2 years and up) who weigh more than 20 kgs [13]. Risdiplam may have harmful effects on the fetus when administered to pregnant women and may also compromise male fertility. Although there are not adequate data from pregnant women receiving risdiplam treatment, data from animal studies demonstrate embryofetal mortality, malformations, and reduced fetal weight. The prescribing information associated with risdiplam, therefore, recommends pregnancy testing to be carried out for females before initiating treatment [95]. 

#### 4.1.7. Combination Therapies

With both AON-based nusinersen treatment and the gene-therapy-based onasemnogene treatment proving to be milestones in finding a cure for SMA, the potential of combination therapy has caught the interest of a few research groups. 

Lee et al. first provided an insight on two patients who were first treated with nusinersen, followed by onasemnogene [96]. Another group studied five patients aged 17–29 months with type 1 SMA who received combination therapy [93]. Four out of five patients received nusinersen before onasemnogene treatment [97]. Elevated liver enzymes were seen in these four patients, with patient 2 exhibiting signs of liver failure, probably related to an immune response because of onasemnogene. Two patients also demonstrated mild thrombocytopenia. Liver enzymes were controlled using corticosteroids. One patient (patient 3), who received onasemnogene treatment before nusinersen, reported no adverse effects. Despite improvements following nusinersen treatment in these patients, the rationale for onasemnogene treatment was the continued need for respiratory support and/or no changes in the bulbar function [97]. The small sample size of the study hinders the comparison between monotherapy and combination therapy. It also remains unclear whether SMN expression levels are boosted by combination therapy or by onasemnogene or nusinersen treatment alone. One study focused on an SMA type 0 patient who had only one copy of *SMN2* [98]. Type 0 is generally very rare since it is rapidly fatal within days to months. The infant received nusinersen treatment at 14 days of age, with a higher CHOP-INTEND score one month later. However, the infant was on tracheostomy for respiratory support and a G-tube for feeding. On day 114, onasemnogene was administered, which also contributed to an increase in the motor function store. Despite small gains in motor function, the treatment course was very complex because of several medical problems [98]. There were a few improvements in the bulbar function, but the treatment did not decrease the level of respiratory support. Cardiac abnormalities and distal necrosis were also quite evident.

A phase II, open-label, multi-center, interventional, exploratory study (JEWELFISH, NCT03032172) is currently evaluating the safety, tolerability, and PK of risdiplam (RO7034067) in infants, children, and adults previously enrolled in Study BP29420 (Moonfish) with the splicing modifier RO6885247 or previously treated with nusinersen or onasemnogene. The participants are expected to receive multiple doses of risdiplam orally once daily for 24 months. Following this period, they will be offered the opportunity to participate in the open-label extension (OLE) study. Extensive and elaborate studies are necessary to strengthen the beneficial evidence, in any form of combination therapies. 

### 4.2. SMN-Independent Therapies

Reldesemtiv (CK2127107), a next-generation fast skeletal muscle troponin activator (FSTA) that slows down the release of calcium from skeletal muscle, was investigated for SMA type II, III, or IV patients (NCT02644668) in a phase II trial [20]. The primary objective of this study was to assess the effects of PD of reldesemtiv on pulmonary and respiratory functions, motor function, and muscle strength. Participants in the trial received either 150 mg or 450 mg twice daily for 8 weeks [99]. A couple of patients from both dosing cohorts reported serious adverse events such as an increase in blood creatine phosphokinase and aspartate aminotransferase and gastrointestinal infections. Other minor adverse events include nausea, vomiting, constipation, fatigue, etc. Patients receiving the higher dose performed better than the lower-dose and placebo cohorts in the 6 min walk test (6MWT) that measures aerobic capacity and endurance. Ambulatory patients in the higher-dose cohort also performed better in the TUG test that measures the time taken for a patient to rise from a chair, walk 3 m, turn back to the chair, and sit down [95]. Patients in the 450 mg cohort also showed an increase in maximal expiratory pressure (MEP). Further studies in patients, including type 1 infants, are necessary to determine the efficacy.

Another study, TOPAZ, is currently assessing the safety and efficacy of SRK-015, a myostatin inhibitor that prevents protease cleavage, prevents myostatin activation, and increases muscle growth and differentiation [20]. This study is currently active and has SMA type II and III patients enrolled. (NCT03921528). BIIB110 is a hybrid activin II receptor (ACTIIR) ligand trap that also targets and sequesters myostatin. This in turn promotes muscle mass and function. It is currently in phase 1 of clinical development. Several other candidates are currently in the preclinical stages that include the NU-p38aMAPK inhibitor by Columbia University [100], second generation AONs by Biogen/Ionis, and small molecules by Calibr and AurimMed Pharma. Further advancements in the development phase will pave the way for clinical trials for these drugs.

## 5. Conclusions

The landscape of SMA therapeutics has changed considerably in the last several years with the FDA approval of DNA- and RNA-targeted therapies. With the discovery of the SMA-causing gene *SMN1*, it became a well-known that the severity of the phenotype is modulated by the number of copies of the paralog gene *SMN2*. Indeed, the determination of the *SMN2* copy number laid the foundation for understanding SMN biology and the SMA phenotype. Advancements in molecular diagnostics, genetic testing, and counseling have contributed to the development of strategies to increase SMN levels in the CNS and tissues body-wide. Exon inclusion therapy using AONs was the first success story following the discovery of the ISS-N1 region in exon 7 of *SMN2*, giving birth to the first FDA-approved AON for SMA in 2016—nusinersen/Spinraza. Gene therapy is another promising therapy to augment SMN levels and has been approved for SMA patients <2 years of age. 

However, several factors need to be considered for both these approved treatments. For example, both these treatments are extremely expensive. Additionally, nusinersen requires repeated intrathecal injections that are often associated with thrombocytopenia and injection-site adverse events (Spinraza prescribing information: reference ID: 4625921). Most significant is that nusinersen must be injected directly into cerebrospinal fluid intrathecally, due to renal toxicity, and therefore can be used to treat only upper motor neurons in the CNS. Although nusinersen is expected to extend the lifespan of people with SMA, treating organs beyond motor neurons will be increasingly important. Findings in SMA mouse models reveal a reduction in spleen size attributed to SMN loss, along with mislocalization of the immune cells in the Smn^2B/−^ model [101]. SMN protein is essential for proper development of lymphoid organs since immune dysregulation contributes to SMA pathogenesis [101,102]. Furthermore, scoliosis or the abnormal curvature of the spine may often make it difficult to administer nusinersen intrathecally. Onasemnogene, an intravenously administered scAAV9-SMN vector, was approved by the FDA in 2019 as a one-time drug for all SMA patients below the age of 2 years. A limited time frame for treatment calls for newborn screening of SMA to ensure early diagnosis and timely treatment. SMA is a progressive muscle-wasting disorder that can be mitigated when treated earlier, as evidenced by several studies. However, the milder forms of SMA only appear in later stages, making genetic screening of infants the most crucial step to ensure the course of treatment for these patients. Although gene therapy provides robust SMN expression throughout the body, the long-term effects of SMN overexpression in patients are unknown. Current clinical trials are evaluating the long-term safety and toxicity effects. Recent studies highlight a gain of toxic function of SMN, especially in the sensorimotor circuit, including the loss of proprioceptive neurons. Nevertheless, gene therapy does provide significant improvements in the motor and respiratory functions in the most vulnerable SMA type 1 patients. The relevance of these findings of neurotoxicity arising from SMN overexpression in mice and patients still needs to be carefully evaluated. Different modes of administration may lead to different levels of SMN expression. Additionally, levels of SMN decline with age in humans, making the time of intervention important. Nevertheless, long-term clinical, electrophysiological, and pathological studies in SMA patients treated with Zolgemsma will aid in the understanding of toxicity associated with SMN overexpression, if any. Risdiplam, a small molecule that acts as an *SMN2* splicing modifier, received its approval in 2020 for all SMA types in patients 2 years of age and up after demonstrating functional improvements in patients. Although it overcomes the necessity for invasive intrathecal injections, it poses a threat to embryofetal health and reproductive health. Despite the correlation of the disease severity with SMN deficiency, there remain missing pieces of the puzzle to clearly understand the pathology and its link to the role of SMN. For example, the actual reason for the susceptibility of motor neurons to SMN deficiency remains unclear. Future studies in vitro and in vivo can help in characterizing the disease mechanism and pave the way for combination therapies that are still being studied in small patient groups. Overall, the prognosis of SMA, in general, is improving because of the developing therapies to augment SMN levels as well as improve muscle strength and function, which ultimately ameliorates the SMA pathology.

## Figures and Tables

**Table 1:** Approved therapies for spinal muscular atrophy.

Treatment Name	Type	Manufacturer	Science	Status
Nusinersen (brand name Spinraza)	Antisense oligonucleotide	Biogen	AON with MOE chemistry targeting *SMN2* ISS-N1 that promotes exon 7 inclusion	Approved by FDA in 2016
Onasemnogene abeparvovec (brand name Zolgensma)	Gene therapy	Novartis, AveXis	scAAV9-SMN under the control of a CBA promoter	Approved by FDA in 2019
Risdiplam (brand name Evrysdi)	Small molecule	Roche	SMN2 splicing modifier administered orally	Approved by FDA in 2020

FDA: Food and Drug Administration; MOE: 2′O-methoxyethyl; scAAV9: self-complementary adeno-associated virus 9; CBA: chicken-β-actin.

**Table 2:** Classification of SMA.

SMA Type	Age of Onset	Maximum Age of Survival	*SMN2* Copy Number	Alternate Name
0	Prenatal	<1 month	1	-
1	0–6 months	<2 years	2	Werdnig–Hoffman disease
2	<18 months	>2 years	3,4	Dubowitz disease
3a (juvenile onset)	18 months–3 years	Adult	3,4	Kugelberg–Welander disease
3b	>3 years	Adult	4	Kugelberg–Welander disease
4	> 21 years	Adult	4–8	-

## Data Availability

Not applicable.

## References

[1] Munsat T.L., Davies K.E. (1992). International SMA consortium meeting. (26–28 June 1992, Bonn, Germany). Neuromuscul. Disord..

[2] Lunn M.R., Wang C.H. (2008). Spinal muscular atrophy. Lancet.

[3] Son H.W., Yokota T. (2018). Recent Advances and Clinical Applications of Exon Inclusion for Spinal Muscular Atrophy. Methods Mol. Biol..

[4] Oskoui M., Kaufmann P. (2008). Spinal muscular atrophy. Neurotherapeutics.

[5] Liu Q., Fischer U., Wang F., Dreyfuss G. (1997). The spinal muscular atrophy disease gene product, SMN, and its associated protein SIP1 are in a complex with spliceosomal snRNP proteins. Cell.

[6] Schrank B., Gotz R., Gunnersen J.M., Ure J.M., Toyka K.V., Smith A.G., Sendtner M. (1997). Inactivation of the survival motor neuron gene, a candidate gene for human spinal muscular atrophy, leads to massive cell death in early mouse embryos. Proc. Natl. Acad. Sci. USA.

[7] Hamilton G., Gillingwater T.H. (2013). Spinal muscular atrophy: Going beyond the motor neuron. Trends Mol. Med..

[8] Burghes A.H., Beattie C.E. (2009). Spinal muscular atrophy: Why do low levels of survival motor neuron protein make motor neurons sick?. Nat. Rev. Neurosci..

[9] Lorson C.L., Hahnen E., Androphy E.J., Wirth B. (1999). A single nucleotide in the SMN gene regulates splicing and is responsible for spinal muscular atrophy. Proc. Natl. Acad. Sci. USA.

[10] Feldkotter M., Schwarzer V., Wirth R., Wienker T.F., Wirth B. (2002). Quantitative analyses of SMN1 and SMN2 based on real-time lightCycler PCR: Fast and highly reliable carrier testing and prediction of severity of spinal muscular atrophy. Am. J. Hum. Genet..

[11] Hoy S.M. (2017). Nusinersen: First Global Approval. Drugs.

[12] Hoy S.M. (2019). Onasemnogene Abeparvovec: First Global Approval. Drugs.

[13] Dhillon S. (2020). Risdiplam: First Approval. Drugs.

[14] Kolb S.J., Kissel J.T. (2011). Spinal muscular atrophy: A timely review. Arch. Neurol..

[15] Dubowitz V. (2009). Ramblings in the history of spinal muscular atrophy. Neuromuscul. Disord..

[16] Munsat T.L. (1991). Workshop report: International SMA collaboration. Neuromuscul. Disord..

[17] Lefebvre S., Burglen L., Reboullet S., Clermont O., Burlet P., Viollet L., Benichou B., Cruaud C., Millasseau P., Zeviani M. (1995). Identification and characterization of a spinal muscular atrophy-determining gene. Cell.

[18] Melki J., Lefebvre S., Burglen L., Burlet P., Clermont O., Millasseau P., Reboullet S., Benichou B., Zeviani M., Paslier D.L. (1994). De novo and inherited deletions of the 5q13 region in spinal muscular atrophies. Science.

[19] Rochette C.F., Gilbert N., Simard L.R. (2001). SMN gene duplication and the emergence of the SMN2 gene occurred in distinct hominids: SMN2 is unique to Homo sapiens. Hum. Genet..

[20] Lefebvre S., Sarret C. (2020). Pathogenesis and therapeutic targets in spinal muscular atrophy (SMA). Arch. Pediatrics.

[21] Liu Q., Dreyfuss G. (1996). A novel nuclear structure containing the survival of motor neurons protein. EMBO J.

[22] Carvalho T., Almeida F., Calapez A., Lafarga M., Berciano M.T., Carmo-Fonseca M. (1999). The spinal muscular atrophy disease gene product, SMN: A link between snRNP biogenesis and the Cajal (coiled) body. J. Cell Biol..

[23] Kolb S.J., Battle D.J., Dreyfuss G. (2007). Molecular functions of the SMN complex. J. Child Neurol..

[24] Burlet P., Huber C., Bertrandy S., Ludosky M.A., Zwaenepoel I., Clermont O., Roume J., Delezoide A.L., Cartaud J., Munnuch A. (1998). The distribution of SMN protein complex in human fetal tissues and its alteration in spinal muscular atrophy. Hum. Mol. Genet..

[25] Singh R.N., Howell M.D., Ottesen E.W., Singh N.N. (2017). Diverse role of survival motor neuron protein. Biochim. Biophys. Acta Gene Regul. Mech..

[26] Coovert D.D., Le T.T., McAndrew P.E., Strasswimmer J., Crawford T.O., Mendell J.R., Coulson S.E., Androphy E.J., Prior T.W., Burghes A.H. (1997). The survival motor neuron protein in spinal muscular atrophy. Hum. Mol. Genet..

[27] Boido M., Vercelli A. (2016). Neuromuscular junctions as key contributors and therapeutic targets in spinal muscular atrophy. Front. Neuroanat..

[28] Kariya S., Park G.H., Maeno-Hichiki Y., Leykekhman O., Lutz C., Arkovitz M.S., Landmesser L.T., Monani U.R. (2008). Reduced SMN protein impairs maturation of the neuromuscular junctions in mouse models of spinal muscular atrophy. Hum. Mol. Gen..

[29] Tisdale S., Van Alstyne M., Simon C.M., Mentis G.Z., Pellizzoni L. (2021). SMN controls neuromuscular junction integrity through U7 snRNP. Biorxiv.

[30] Singh N.K., Singh N.N., Androphy E.J., Singh R.N. (2006). Splicing of a critical exon of human Survival Motor Neuron is regulated by a unique silencer element located in the last intron. Mol. Cell. Biol..

[31] Singh N.N., Shishimorova M., Cao L.C., Gangwani L., Singh R.N. (2009). A short antisense oligonucleotide masking a unique intronic motif prevents skipping of a critical exon in spinal muscular atrophy. RNA Biol..

[32] Ottesen E.W. (2017). ISS-N1 makes the First FDA-approved Drug for Spinal Muscular Atrophy. Transl. Neurosci..

[33] Hua Y., Vickers T.A., Okunola H.L., Bennett C.F., Krainer A.R. (2008). Antisense masking of an hnRNP A1/A2 intronic splicing silencer corrects SMN2 splicing in transgenic mice. Am. J. Hum. Genet..

[34] Williams J.H., Schray R.C., Patterson C.A., Ayitey S.O., Tallent M.K., Lutz G.J. (2009). Oligonucleotide-mediated survival of motor neuron protein expression in CNS improves phenotype in a mouse model of spinal muscular atrophy. J. Neurosci..

[35] Hua Y., Sahashi K., Hung G., Rigo F., Passini M.A., Bennett C.F., Krainer A.R. (2010). Antisense correction of SMN2 splicing in the CNS rescues necrosis in a type III SMA mouse model. Genes Dev..

[36] Passini M.A., Bu J., Richards A.M., Kinnecom C., Sardi S.P., Stanek L.M., Hua Y., Rigo F., Mateson J., Hung G. (2011). Antisense oligonucleotides delivered to the mouse CNS ameliorate symptoms of severe spinal muscular atrophy. Sci. Transl. Med..

[37] Hua Y., Sahashi K., Rigo F., Hung G., Horev G., Bennett C.F., Krainer A.R. (2011). Peripheral SMN restoration is essential for long-term rescue of a severe spinal muscular atrophy mouse model. Nature.

[38] Sardone V., Zhou H., Muntoni F., Ferlini A., Falzarano M.S. (2017). Antisense Oligonucleotide-Based Therapy for Neuromuscular Disease. Molecules.

[39] Summerton J., Weller D. (1997). Morpholino antisense oligomers: Design, preparation, and properties. Antisense Nucleic Acid Drug Dev..

[40] Summerton J.E. (2017). Invention and Early History of Morpholinos: From Pipe Dream to Practical Products. Methods Mol. Biol..

[41] Porensky P.N., Mitrpant C., McGovern V.L., Bevan A.K., Foust K.D., Kaspar B.K., Wilton S.D., Burghes A.H.M. (2012). A single administration of morpholino antisense oligomer rescues spinal muscular atrophy in mouse. Hum. Mol. Genet..

[42] Zhou H., Janghra N., Mitrpant C., Dickinson R.L., Anthony K., Price L., Eperon I.C., Wilton S.D., Morgan J., Muntoni F. (2013). A novel morpholino oligomer targeting ISS-N1 improves rescue of severe spinal muscular atrophy transgenic mice. Hum. Gene Ther..

[43] Hsieh-Li H.M., Chang J.G., Jong Y.J., Wu M.H., Wang N.M., Tsai C.H., Li H. (2000). A mouse model for spinal muscular atrophy. Nat. Genet..

[44] Nizzardo M., Simone C., Salani S., Ruepp M.D., Rizzo F., Ruggieri M., Zanetta C., Brajkovic S., Moulton H.M., Muehlemann O. (2014). Effect of combined systemic and local morpholino treatment on the spinal muscular atrophy Delta7 mouse model phenotype. Clin. Ther..

[45] Anwar S., Yokota T. (2020). Golodirsen for Duchenne muscular dystrophy. Drugs Today.

[46] Roshmi R.R., Yokota T. (2019). Viltolarsen for the treatment of Duchenne muscular dystrophy. Drugs Today.

[47] Ramsey J.D., Flynn N.H. (2015). Cell-penetrating peptides transport therapeutics into cells. Pharmacol. Ther..

[48] Frankel A.D., Pabo C.O. (1988). Cellular uptake of the tat protein from human immunodeficiency virus. Cell.

[49] Park J., Ryu J., Kim K.A., Lee H.J., Bahn J.H., Han K., Choi E.Y., Lee K.S., Kwon H.Y., Choi S.Y. (2002). Mutational analysis of a human immunodeficiency virus type 1 Tat protein transduction domain which is required for delivery of an exogenous protein into mammalian cells. J. Gen. Virol.

[50] Betts C., Saleh A.F., Arzumanov A.A., Hammond S.M., Godfrey C., Coursindel T., Gait M.J., Wood M.J.A. (2012). Pip6-PMO, A New Generation of Peptide-oligonucleotide Conjugates with Improved Cardiac Exon Skipping Activity for DMD Treatment. Mol. Ther. Nucleic Acids.

[51] Du L., Kayali R., Bertoni C., Fike F., Hu H., Iversen P.L., Gatti R.A. (2011). Arginine-rich cell-penetrating peptide dramatically enhances AMO-mediated ATM aberrant splicing correction and enables delivery to brain and cerebellum. Hum. Mol. Genet..

[52] Betts C.A., Wood M.J. (2013). Cell penetrating peptide delivery of splice directing oligonucleotides as a treatment for Duchenne muscular dystrophy. Curr. Pharm. Des..

[53] Boisguerin P., Deshayes S., Gait M.J., O’Donovan L., Godfrey C., Betts C.A., Wood M.J.A., Lebleu B. (2015). Delivery of therapeutic oligonucleotides with cell penetrating peptides. Adv. Drug Deliv. Rev..

[54] Jarver P., O’Donovan L., Gait M.J. (2014). A chemical view of oligonucleotides for exon skipping and related drug applications. Nucleic Acid Ther..

[55] Yin H., Betts C., Saleh A.F., Ivanova G.D., Lee H., Seow Y., Kim D., Gait M.J., Wood M.J.A. (2010). Optimization of peptide nucleic acid antisense oligonucleotides for local and systemic dystrophin splice correction in the mdx mouse. Mol. Ther..

[56] Yin H., Moulton H.M., Betts C., Merritt T., Seow Y., Ashraf S., Wang Q., Boutilier J., Wood M.J.A. (2010). Functional rescue of dystrophin-deficient mdx mice by a chimeric peptide-PMO. Mol. Ther..

[57] Yin H., Moulton H.M., Seow Y., Boyd C., Boutilier J., Iverson P., Wood M.J.A. (2008). Cell-penetrating peptide-conjugated antisense oligonucleotides restore systemic muscle and cardiac dystrophin expression and function. Hum. Mol. Genet..

[58] Hammond S.M., Hazell G., Shabanpoor F., Saleh A.F., Bowerman M., Sleigh J.N., Meijboom K.E., Zhou H., Muntoni F., Talbot K. (2016). Systemic peptide-mediated oligonucleotide therapy improves long-term survival in spinal muscular atrophy. Proc. Natl. Acad. Sci. USA.

[59] Osorio F.G., Navarro C.L., Cadinanos J., Lopez-Mejia C., Quiros P.M., Bartoli C., Rivera J., Tazi J., Guzman G., Varela I. (2011). Splicing-directed therapy in a new mouse model of human accelerated aging. Sci. Transl. Med..

[60] Southwell A.L., Kordasiewicz H.B., Langbehn D., Skotte N.H., Parsons M.P., Villanueva E.B., Caron N.S., Ostergaard M.E., Anderson L.M., Xie Y. (2018). Huntingtin suppression restores cognitive function in a mouse model of Huntington’s disease. Sci. Transl. Med..

[61] Aslesh T., Yokota T. (2020). Development of Antisense Oligonucleotide Gapmers for the Treatment of Huntington’s Disease. Methods Mol. Biol..

[62] Kalbfuss B., Mabon S.A., Misteli T. (2001). Correction of alternative splicing of tau in frontotemporal dementia and parkinsonism linked to chromosome. J. Biol. Chem..

[63] Lagier-Tourenne C., Baughn M., Rigo F., Sun S., Liu P., Li H.R., Jiang J., Watt A.T., Chun S., Katz M. (2013). Targeted degradation of sense and antisense C9orf72 RNA foci as therapy for ALS and frontotemporal degeneration. Proc. Natl. Acad. Sci. USA.

[64] Corey D.R. (2017). Nusinersen, an antisense oligonucleotide drug for spinal muscular atrophy. Nat. Neurosci..

[65] Goodkey K., Aslesh T., Maruyama R., Yokota T. (2018). Nusinersen in the Treatment of Spinal Muscular Atrophy. Methods Mol. Biol..

[66] Chiriboga C.A., Swoboda K.J., Darras B.T., Iannaccone S.T., Montes J., Vivo D.C.D., Norris D.A., Bennett C.F., Bishop K.M. (2016). Results from a phase 1 study of nusinersen (ISIS-SMN(Rx)) in children with spinal muscular atrophy. Neurology.

[67] Finkel R.S., Chiriboga C.A., Vajsar J., Day J.W., Montes J., Vivo D.C.D., Yamashita M., Rigo F., Hung G., Schneider E. (2016). Treatment of infantile-onset spinal muscular atrophy with nusinersen: A phase 2, open-label, dose-escalation study. Lancet.

[68] De Vivo D.C., Bertini E., Swoboda K.J., Hwu W.L., Crawford T.O., Finkel R.S., Kirschner J., Kuntz N.L., Parsons J.A., Ryan M.M. (2019). Nusinersen initiated in infants during the presymptomatic stage of spinal muscular atrophy: Interim efficacy and safety results from the Phase 2 NURTURE study. Neuromuscul. Disord..

[69] Finkel R.S., Chiriboga C.A., Vajsar J., Day J.W., Montes J., Vivo D.C.D., Yamashita M., Rigo F., Hung G., Schneider E. (2021). Treatment of infantile-onset spinal muscular atrophy with nusinersen: Final report of a phase 2, open-label, multicentre, dose-escalation study. Lancet Child Adolesc. Health.

[70] Sheikh O., Yokota T. (2021). Restoring Protein Expression in Neuromuscular Conditions: A Review Assessing the Current State of Exon Skipping/Inclusion and Gene Therapies for Duchenne Muscular Dystrophy and Spinal Muscular Atrophy. BioDrugs.

[71] Wurster C.D., Winter B., Wollinsky K., Ludolph A.C., Uzelac Z., Witzel S., Schocke M., Schneider R., Kocak T. (2019). Intrathecal administration of nusinersen in adolescent and adult SMA type 2 and 3 patients. J. Neurol..

[72] Sansone V.A., Pirola A., Albamonte E., Pane M., Lizio A., D’Amico A., Catteruccia M., Cutrera R., Bruno C., Pedemonte M. (2020). Respiratory Needs in Patients with Type 1 Spinal Muscular Atrophy Treated with Nusinersen. J. Pediatrics.

[73] Sheng L., Rigo F., Bennett C.F., Krainer A.R., Hua Y. (2020). Comparison of the efficacy of MOE and PMO modifications of systemic antisense oligonucleotides in a severe SMA mouse model. Nucleic Acids Res..

[74] Foust K.D., Wang X., McGovern V.L., Braun L., Bevan A.K., Haidet A.M., Le T.T., Morales P.R., Rich M.M., Burghes A.H.M. (2010). Rescue of the spinal muscular atrophy phenotype in a mouse model by early postnatal delivery of SMN. Nat. Biotechnol..

[75] Dominguez E., Marais T., Chatauret N., Benkhelifa-Ziyyat S., Duque S., Ravassard P., Carcenac R., Astord S., Pereira de Moura A., Voit T. (2011). Intravenous scAAV9 delivery of a codon-optimized SMN1 sequence rescues SMA mice. Hum. Mol. Genet..

[76] Meyer K., Ferraiuolo L., Schmelzer L., Braun L., McGovern V., Likhite S., Michels O., Govoni A., Fitzgerald J., Morales P. (2015). Improving single injection CSF delivery of AAV9-mediated gene therapy for SMA: A dose-response study in mice and nonhuman primates. Mol. Ther..

[77] Armbruster N., Lattanzi A., Jeavons M., Wittenberghe L.V., Gjata B., Marais T., Martin S., Vignaud A., Voit T., Mavilio F. (2016). Efficacy and biodistribution analysis of intracerebroventricular administration of an optimized scAAV9-SMN1 vector in a mouse model of spinal muscular atrophy. Mol. Ther. Methods Clin. Dev..

[78] Mendell J.R., Al-Zaidy S., Shell R., Arnold W.D., Rodino-Klapac L.R., Prior T.W., Lowes L., Alfano L., Berry K., Church K. (2017). Single-Dose Gene-Replacement Therapy for Spinal Muscular Atrophy. N. Engl. J. Med..

[79] Lowes L.P., Alfano L.N., Arnold W.D., Shell R., Prior T.W., McColly M., Lehman K.J., Church K., Sproule D.M., Nagendran S. (2019). Impact of Age and Motor Function in a Phase 1/2A Study of Infants With SMA Type 1 Receiving Single-Dose Gene R.Replacement Therapy. Pediatrics Neurol..

[80] Al-Zaidy S., Pickard A.S., Kotha K., Alfano L.N., Lowes L., Paul G., Church K., Lehman K., Sproule D.M., Dabbous O. (2019). Health outcomes in spinal muscular atrophy type 1 following AVXS-101 gene replacement therapy. Pediatrics Pulmonol..

[81] Day J.W., Chiriboga C., Crawford T.O., Darras B.T., Finkel R.S., Connolly A.M., Iannaccone S.T., Kuntz N.L., Pena L.D.M., Schultz M. (2019). AVXS-101 gene replacement therapy (GRT) for spinal muscular atrophy type 1 (SMA1): Pivotal phase 3 study (STR1VE) update. Am. J. Respir. Crit. Care Med..

[82] Agency E.M. (2020). Zolgensma: European Public Assessment Report. https://www.ema.europa.eu/en/documents/assessment-report/zolgensma-epar-public-assessment-report_en.pdf.

[83] Van Alstyne M., Tattoli I., Delestree N., Recinos Y., Workman E., Shihabuddin L.S., Zhang C., Mentis G.Z., Pellizzoni L. (2021). Gain of toxic function by long-term AAV9-mediated SMN overexpression in the sensorimotor circuit. Nat. Neurosci..

[84] Hinderer C., Katz N., Buza E.L., Dyer C., Goode T., Bell P., Richman L.K., Wilson J.M. (2018). Severe Toxicity in Nonhuman Primates and Piglets Following High-Dose Intravenous Administration of an Adeno-Associated Virus Vector Expressing Human SMN. Hum. Gene Ther..

[85] Naryshkin N.A., Weetall M., Dakka A., Narasimhan J., Zhao X., Feng Z., Ling K.K.Y., Karp G.M., Qi H., Woll M.G. (2014). Motor neuron disease. SMN2 splicing modifiers improve motor function and longevity in mice with spinal muscular atrophy. Science.

[86] Woll M.G. (2013). Compounds for Treating Spinal Muscular Atrophy.

[87] Lee C.-S., Karp G.M., Koyama H., Ratni H. (2013). Compounds for Treating Spinal Muscular Atrophy.

[88] Ratni H., Karp G.M., Weetall M., Naryshkin N.A., Paushkin S.V., Chen K.S., McCarthy K.D., Qi H., Turpoff A., Woll M.G. (2016). Specific Correction of Alternative Survival Motor Neuron 2 Splicing by Small Molecules: Discovery of a Potential Novel Medicine To Treat Spinal Muscular Atrophy. J. Med. Chem..

[89] Calder A.N., Androphy E.J., Hodgetts K.J. (2016). Small Molecules in Development for the Treatment of Spinal Muscular Atrophy. J. Med. Chem..

[90] Wadman R.I., van der Pol W.L., Bosboom W.M., Asselman F.L., Berg L.H.V.D., Iannaccone S.T., Vrancken A.F. (2020). Drug treatment for spinal muscular atrophy types II and III. Cochrane Database Syst Rev..

[91] Tiziano F.D., Lomastro R., Abiusi E., Pasanisi M.B., Pietro L.D., Fiori S., Baranello G., Angelini C., Soraru G., Gaiani A. (2019). Longitudinal evaluation of SMN levels as biomarker for spinal muscular atrophy: Results of a phase IIb double-blind study of salbutamol. J. Med. Genet..

[92] Ratni H., Ebeling M., Baird J., Bendels S., Bylund J., Chen K.S., Denk N., Feng Z., Green L., Guerard M. (2018). Discovery of Risdiplam, a Selective Survival of Motor Neuron-2 (SMN2) Gene Splicing Modifier for the Treatment of Spinal Muscular Atrophy (SMA). J. Med. Chem..

[93] Baranello G., Darras B.T., Day J.W., Deconinck N., Klein A., Masson R., Mercuri E., Rose K., El-Khairi M., Gerber M. (2021). Risdiplam in Type 1 Spinal Muscular Atrophy. N. Engl. J. Med..

[94] Servais L.B.G., Masson R., Mazukiewicz-Beldzinska M., Rose K., Vlodavets D., Xiong H., Zanotelli E., El-Khairi M., Fuerst-Recktenwald S., Gerber M. (2020). FIREFISH Part 2: Efficacy and safety of risdiplam (RG7916) in infants with type 1 spinal muscular atrophy (SMA). Neurology.

[95] Kakazu J., Walker N.L., Babin K.C., Trettin K.A., Lee C., Sutker P.B., Kaye A.M., Kaye A.D. (2021). Risdiplam for the use of spinal muscular atrophy. Orthop. Rev. Pavia.

[96] Lee B.H., Collins E., Lewis L., Guntrum D., Eichinger K., Voter K., Abdel-Hamid H.Z., Ciafaloni E. (2019). Combination therapy with nusinersen and AVXS-101 in SMA type. Neurology.

[97] Harada Y., Rao V.K., Arya K., Kuntz N.L., DiDonato C.J., Napchan-Pomerantz G., Agarwal A., Stefans V., Katsuno M., Veerapandiyan A. (2020). Combination molecular therapies for type 1 spinal muscular atrophy. Muscle Nerve.

[98] Matesanz S.E., Curry C., Gross B., Rubin A.I., Linn R., Yum S.W., Kichula E.A. (2020). Clinical Course in a Patient With Spinal Muscular Atrophy Type 0 Treated With Nusinersen and Onasemnogene Abeparvovec. J. Child Neurol..

[99] Reldesemtiv (Formerly CK-2127107). https://smanewstoday.com/ck-2127107-ck-107/.

[100] (2018). The Role of p38 MAPK Activation in Spinal Muscular Atrophy (Pellizzoni, L).

[101] Deguise M.O., De Repentigny Y., McFall E., Auclair N., Sad S., Kothary R. (2017). Immune dysregulation may contribute to disease pathogenesis in spinal muscular atrophy mice. Hum. Mol. Genet..

[102] Khairallah M.T., Astroski J., Custer S.K., Androphy E.J., Franklin C.L., Lorson C.L. (2017). SMN deficiency negatively impacts red pulp macrophages and spleen development in mouse models of spinal muscular atrophy. Hum. Mol. Genet..

